# Assessment of social justice dimensions in young adults: The contribution of the belief in a just world and social dominance orientation upon its rising

**DOI:** 10.3389/fpsyg.2022.997423

**Published:** 2022-11-03

**Authors:** Edgardo Etchezahar, Alicia Barreiro, Miguel Ángel Albalá Genol, Antonio Francisco Maldonado Rico

**Affiliations:** ^1^Department of Social Psychology, Faculty of Psychology, University of Buenos Aires, Buenos Aires, Argentina; ^2^Department of Evolutionary Psychology and Education, Universidad Autónoma de Madrid, Madrid, Spain; ^3^Faculty of Education, International University of Valencia (VIU), Valencia, Spain; ^4^Consejo Nacional de Investigaciones Científicas y Técnicas, CONICET, Buenos Aires, Argentina; ^5^Department of Educational Psychology, Facultad Latinoamericana de Ciencias Sociales, FLACSO, Buenos Aires, Argentina

**Keywords:** social justice, representation, recognition, redistribution, belief in a just world, social dominance

## Abstract

The aim of the study was to analyze the psychometric properties of the Social Justice Scale, composed by Representation, Recognition, and Redistribution dimensions. Likewise, the contribution of social dominance and the belief in a just world in each dimension were analyzed. A total of 471 young adults residing in Madrid participated in the online preliminary study, with an age range of 18–42 years with different genders (74.1% defined themselves as female). The main results indicated adequate psychometric properties for Social Justice Scale through its three dimensions. In addition, we observed that both social dominance and belief in a just world might be psychosocial variables that modulate the levels of social justice. The main findings of the research and need for replication in future studies are discussed.

## Introduction

In recent decades, discussions on the concept and relevance of Social Justice have reached a great interest, overflowing the limits of Political Philosophy and the Theory of Law (e.g., Taylor, 1993; Habermas, 1998; Rawls, 2001; Sen, 2009) and extending to the broader field of Social Sciences (Young, 2004; Fraser, 2008; Nussbaum, 2012). This interest was accentuated by reflections on global citizenships and migratory processes that, together with deep economic, social, and legal inequalities, constantly challenge the nineteenth-century ideal of homogeneous identity construction (Westheimer, 2015). Despite the equality values, recognition, and respect for differences roundly consolidated after the atrocities committed in the first half of the twentieth century (El Navas, 1997), current societies are plagued by inequalities, exclusion, and discrimination, whether in terms of gender, social class, culture or sexual preference (Jacott et al., 2014). Issues about structural injustices and their approaches in nowadays globalized societies have been debated, but not comprehensively from the perspective of power differences as applied to social psychological perspectives on justice (Liu and Pratto, 2018; Plenty, 2018).

Liberal perspectives focused social justice analysis on inequalities resulting from material and cultural goods distribution or other types of resources (Rawls, 2001). Thus, they proposed that equity in social conditions makes a fair distribution of social goods and resources possible. In addition, according to Nussbaum (Nussbaum, 2012), a fundamental requirement to achieve Social Justice is respect for human dignity, which implies the obligation that citizenship be situated above a broad (and specific) minimum threshold in at least 10 central capacities. Different authors noted that because of the profound cultural differences in contemporary societies, such equity would be impossible without recognizing the particularities of the diverse social groups (e.g., Young, 1990; Taylor, 1993; Fraser, 1995). Besides these lines, for such recognition to be assertive -avoiding perpetuating inequality-, the groups involved must participate in decision-making on issues affecting disparate aspects of their lives (Miller, 1999; Young, 2004; Fraser, 2008).

### Three-dimensional model of social justice: Redistribution, recognition and representation

Based on different authors (Murillo et al., 2014; Hernández-Castilla and Hidalgo Farran, 2015), in order to assess social justice it is essential to base on a multidimensional concept (focused on at least three types of injustices: economic, cultural and political) considering the great diversity of existing injustices. Within this context, (Fraser, 2008) proposed social justice should be analyzed by involving to three constitutively related dimensions: redistribution of resources (not only economic but also educational, social, health, etc.), recognition of diversity (whether of identities, genders, cultures, etc.) and representation (participation in decision-making that affects people’s lives, both in the redistribution of resources and multiple identities recognition). It is possible to identify injustices affecting these three dimensions in any society (Murillo and Hernández-Castilla, 2011). In the present study, these considerations were addressed in order to design a Social Justice scale that was as wide as possible and adapted to current injustices and problems. Firstly, according to Fraser (Fraser, 2008) based on a classic perspective of the concept, there is Redistribution or Economic Justice (Rawls, 2001; Sen, 2009), which suggests the need for a just distribution of goods and material resources, as well as distribution at the cultural level. Secondly, the dimension of Recognition or Cultural Justice (Fraser and Honneth, 2006) highlights the need for sociocultural respect towards all people, as well as to value human diversity and promote fair relationships. This perspective encourages an absence of social and cultural domination, giving visibility and recognition to minorities that have been historically excluded for different reasons. Thirdly, the notion of Representation or Political Justice (Young, 1990) attempts to give people the option to participate in society in an active and equal way. Therefore, the present study is aimed to validate an instrument based on a philosophical conception of social justice closer to the social sciences, in order to empirically contrast said paradigm.

For example, in Spanish society, economic and resource inequality between social groups is profound. This situation of social unfairness is evident when considering that Spain exhibits one of the lowest equality indicators in the European Union, along with Bulgaria, Greece, and Lithuania (European Union: European Commission, Report from the Commission to the European Parliament and the Council: Progress Report on the Implementation of the Hotspot Approach in Greece, 2016). While in most of the countries that make up the European Union the top 20% of people with the highest salaries earn five times the 20% who earn the least, in Spain this ratio is six and a half times above average. Additionally, in terms of recognition, women continue to suffer discrimination both at work and personally, as reported in the public sphere (Cepeda González, 2017). Likewise, the situation of minority groups in Spanish society, like those with other cultural identities resulting from the incorporation of migrants, or the scarce tradition of respect for their linguistic diversity within the State, is also worrying. Other recent studies show significant levels of prejudice (both subtle and blatant) towards minority groups such as, for example, the Roma people (e.g., Cuadrado et al., 2016; Etchezahar et al., 2016). Moreover, as mentioned earlier and following (Fraser and Honneth, 2006; Fraser, 2008), resource distribution and recognition are expressed in the possibility of social groups’ involvement, beyond political representation. In the Spanish context, the situation concerning this dimension is also worrying. Several studies indicate that young people reject and take distance from partisan politics, preferring new forms of community-based citizen participation, whether in NGOs or different local initiatives (Parés, and y Subirats, J., 2016). In this regard, we must underline democratic systems depend on a politically active citizenry (Bierle and Cayford, 2002; Fischer, 2018). Therefore, the common framework of justice around citizenship can be seen as a psychosocial process that seeks to ensure social reconciliation in societies which have been fragmented and seek to improve democracy (Basabe and Páez, 2021).

Despite the social relevance of social justice-related issues and its broad intellectual tradition, few precedent studies have adopted a psychological perspective. The analysis of justice-related judgments boasts a vast tradition, from the pioneering work of (Piaget, 1976/1932), through the contributions of (Kohlberg, 1971), to contemporary constructivist proposals (Barreiro et al., 2019; Elenbaas et al., 2020). Such works were not devoted to analyzing the representations that comprise how people think about social justice, considering the three dimensions proposed by Fraser. However, numerous instruments have been designed to assess variables that guide or maintain certain injustices, through the attribution of responsibility and/or the justification of social domination. In relation to social justice, several of these instruments evaluate aspects proposed by (5), but they do so in isolation and focusing on the situation of injustice or the assessment of its consequences. In order to evaluate the representations of social justice, which can respond as predictive or consistent variables with those already studied, it is necessary to develop a new instrument that fully responds to social demands. In this way, it is intended not only to evaluate the construct of social justice in a multidimensional way, but also to know the scope of each factor that composes it. Therefore, to date, we lack valid and reliable instruments specifically designed to assess how people represent social justice and how these representations relate to psychosocial variables widely studied as responsible for maintaining social inequalities, such as belief in a just world and social dominance orientation (Etchezahar and Brussino, 2015; Barreiro et al., 2019).

### The evaluation of social justice and other psychosocial variables involved

The assessment of Social Justice representations has a first scale based approached in the study conducted by (Enterline et al., 2008), who built a 12-item scale called “Learning to Teach Beliefs about Social Justice” (LTSJ-B). This technique consists of a survey containing indicators on the perception, expectations, and beliefs of U.S. teachers about retributive justice in the teaching process (Enterline et al., 2008). Some years after the development of this assessment, (Ginns et al., 2015) tested it in Australia, obtaining adequate psychometric results. While considered groundbreaking in the analysis of social justice, this study focuses only on one aspect of the construct: the distribution of human and economic resources.

On the other hand, following theory of planned behavior, (Torres-Harding et al., 2012) develops a social justice evaluation and used it with practitioners, students, professors, and different community members. The authors consider social justice as a set of values or beliefs referring to equitable access to social resources and protection of human resources, which need to be evaluated in four dimensions: attitudes towards social justice, perception of behavioral control, subjective norm, and behavioral intentions. From their perspective, society should work towards the empowerment of disadvantaged people. Their analysis attempts to take a further step in understanding how certain attitudes towards social justice are closely related to direct action. Like the (Enterline et al., 2008) assessment, the (Torres-Harding et al., 2012) study focuses on one aspect of social justice: equity in terms of goods and resources distribution.

In contrast (Murillo et al., 2014), empirically analyzed the different dimensions of social justice according to (Fraser, 2008): redistribution, recognition, and representation. The authors developed a 16-item scale, elaborated from three dimensions: social justice in society, education with social justice, and commitment to social justice. They obtained adequate reliability and validity indicators in a sample of Spanish students and teachers. As indicated by the authors, this scale enables exploring personal and social factors affecting social justice, such as education, values, and personal experiences in the three dimensions of the construct. However, it does not discriminate the elements of representation, recognition, and redistribution.

Along these lines (Murillo and Hernández-Castilla, 2011), developed a Social Justice Questionnaire comprising a set of moral dilemmas to assess the three dimensions of social justice in teachers and students of primary and secondary education. The questionnaire consists of a set of dilemmas with different response options, created according to their proximity-distance to the social justice dimensions proposed by (Fraser, 2008) (redistribution, recognition, and representation). Participants must choose which option they consider represents their way of thinking. This technique is the first to advance in analyzing the three social justice dimensions in a specific way. Moreover, based on the study of the dilemmas included, different works strengthened this line of research, showing valid evidence (Jacott and Maldonado, 2012). Because how were they built, many of these dilemmas share different dimensions of social justice, hence, it is difficult to analyze individually in each participant the dimension levels of representation, redistribution, and recognition. According to the authors (Jacott et al., 2014), constructing dilemmas representing each dimension secludedly is complex. For example, if we analyze: “a school has a playground where a girl with a wheelchair cannot play, what should the school do?” with the response options: (1) The school must adapt the playground so the girl with a wheelchair can play like the rest, (2) The school must adapt part of the playground to give the girl a place to play or (3) The school needs to evaluate whether the costs involved in adapting the playground are a priority. On the one hand, the redistribution of the school’s resources is at stake; however, diversity is also explicitly recognized in terms of the possibilities of the different actors involved.

There is an extensive tradition dedicated to study psychosocial variables that favor the maintenance of social inequality. These types of variables have shown to be related to the measures that directly or indirectly justify the injustices of the system in which they occur (Vargas-Salfate et al., 2018). These include studies on the belief in a just world (Lerner, 1980; Lerner and Clayton, 2011). This belief responds to the need to live in a stable and predictable environment, that makes future planning possible, and protects against injustice. Thus, the belief in a just world is at the basis of meritocratic social systems and favors the maintenance of social order (Jost and Hunyady, 2005) by denying social injustices (Kay and Jost, 2003; Wolfradt and Dalbert, 2003; Sutton and Douglas, 2005). Therefore, this belief would be the outcome of an ideological appropriation process (Barreiro and Castorina, 2015). Another psychosocial variable explored to explain the maintenance of inequalities is social dominance orientation (Pratto et al., 1994). The theory of social dominance proposes a psychological mechanism based on the tendency to establish and maintain social hierarchies, conceiving certain groups as superior and others as inferior (Sidanius and Pratto, 1999; Sidanius et al., 2000). From this perspective, subordinated people should have at their disposal representations of social justice to empower them to meet their needs and to equalize their power with that of other groups (Liu and Khan, 2021). It is worth noting that, according to (Lambert et al., 1998), the social dominance and the belief in a just world make up a belief system with negative consequences for society that underestimates and blames the victims of injustice. We hypothesize that social dominance and the belief in a just world are the ideological backgrounds of social justice.

In the first place, this paper proposes the development of a social justice representation assessment aimed at discriminating its three dimensions (Representation, Recognition, and Redistribution) and studying whether the differences depend on the gender of the participants. Secondly, we analyze if the belief in a just world and social dominance are predictive of the social justice dimensions.

## Materials and methods

### Sample

The study included 471 young adults of Madrid (Spain), who answered an online questionnaire, using social media advertising targeted by gender (74.1% were women and 25.9% men), age range of 18 to 42 years (*M* = 19.88; *SD* = 2.74) and socioeconomic level (4,25% belonged to the lower class, 23,15% to lower middle class, 66,03% to middle and 6.59% to upper middle), according the national census (INE, 2019). The sampling was non-probabilistic (convenience sampling) (Pérez López, 2005; Hernández Sampieri et al., 2014).

### Instruments

We used a self-administered evaluation instrument comprising the following scales:

#### Social justice scale

For the construction of the Social Justice Scale, we followed the first methodological steps recommended by the International Test Commission (ITC) (Hambleton et al., 2005). First, we developed three sets of items based on each social justice dimension, ensuring their meaning remained independent from the rest of the dimensions. Subsequently, we proceeded to perform an inter-judge evaluation, using a list of items and the abbreviated definition of each dimension of social justice (recognition, redistribution, and representation) and asked them to indicate which dimension or dimensions of social justice each item represented. From the evaluation of the three judges, we reached 30 items (10 for each dimension) with 100% agreement regarding each one belonging to its factor, independently of the remaining items by criteria of the three judges. After the data analysis presented in the next section, we arrived at a final scale of 18 items (6 for each dimension) (Table 1). The scale response format was Likert-type with five agreement levels, *1 = “Strongly agree” and 5 = “Strongly disagree.”*

**Table 1:** Descriptive analysis of the social justice scale items.

	*M*	*SD*	*r_jx_*	α.-x	1	2	3
**Redistribution (α = 0.73)**							
1. Los países más ricos deberían transferir una parte significativa de sus recursos a los países más pobres.	3.92	1.09	0.429	0.702	**0.753**	0.147	0.023
*The richest countries should transfer a significant part of their resources to the poorest countries.*							
4. Las personas con mayor riqueza deberían pagar más impuestos que los que menos tienen.	4.11	1.19	0.449	0.696	**0.741**	0.175	0.081
*People with more wealth should pay more taxes than those with less.*							
7. Debería regularse por ley que los trabajadores de una empresa participen en las ganancias.	3.83	1.09	0.481	0.688	**0.687**	0.274	0.053
*It should be regulated by law that the workers of a company participate in its profits.*							
10. Todas las personas desempleadas deberían recibir ayuda económica del Estado.	3.74	1.22	0.473	0.689	**0.679**	0.102	0.26
*All unemployed people should receive financial aid from the State.*							
13. La jubilación debe concederse independientemente de si la persona ha realizado aportes.	3.73	1.27	0.387	0.717	**0.654**	0.061	0.223
*Retirement pension should be granted regardless the person has made contributions.*							
16. Las familias más pobres deberían recibir más dinero del Estado que las que tienen mayores recursos.	3.74	1.19	0.575	0.658	**0.551**	0.157	0.133
*The poorest families should receive more money from the State than those with more resources.*							
**Recognition (α = 0.76)**							
2. El Estado debería aumentar la cantidad de profesores inmigrantes en las escuelas públicas.	3.05	0.95	0.401	0.713	0.057	**0.783**	0.112
*The State should increase the number of immigrant teachers in public schools.*							
5. En el Congreso debería haber representantes de todas las minorías étnicas o culturales.	4.14	0.99	0.384	0.717	0.056	**0.722**	0.232
*It should be representatives of all ethnic or cultural minorities in Congress.*							
8. Todos los habitantes de un país deben tener derecho a recibir la nacionalidad, aunque no hayan nacido allí.	3.81	1.15	0.502	0.684	0.189	**0.682**	0.202
*All the inhabitants of a country should have the right to receive nationality, even if they were not born there.*							
11. Los inmigrantes que viven en este país tendrían que poder mantener sus costumbres y cultura.	3.79	1.17	0.403	0.715	0.143	**0.633**	0.176
*Immigrants living in this country should be able to maintain their customs and culture.*							
14. Los inmigrantes deben tener el mismo derecho al voto que los ciudadanos locales.	3.87	1.26	0.573	0.66	0.098	**0.605**	0.018
*Immigrants should have the same right to vote as local citizens.*							
17. A las personas migrantes se les deben reconocer los mismos derechos que a las personas que tienen la nacionalidad de ese país.	4.15	1.03	0.55	0.672	0.004	**0.504**	0.099
*Migrants should be granted with the same rights as people who have the nationality of that country.*							
**Representation (α = 0.65)**							
3. Las leyes aprobadas sin amplia mayoría en el Congreso, deberían ser ratificadas o rechazadas por el voto ciudadano.	3.91	1.08	0.401	0.599	0.166	0.113	0.738
*Laws approved without a large majority in Congress should be ratified or rejected by the citizen vote.*							
6. Los ciudadanos deberían decidir cómo se distribuye el presupuesto de un gobierno.	3.68	1.25	0.451	0.578	0.088	0.123	0.696
*Citizens should decide how a government budget is distributed.*							
9. No es útil realizar consultas ciudadanas para la elaboración de leyes o la toma de decisiones políticas.	1.73	1.12	0.204	0.67	0.271	0.083	0.683
*It is not useful to carry out citizen consultations for the elaboration of laws or the making of political decisions.*							
12. Los estudiantes deberían tener una participación activa sobre cómo se gestiona el aprendizaje en las aulas.	3.98	1.06	0.342	0.62	0.207	0.223	0.677
*Students should have an active participation in how learning is managed in the classroom.*							
15. Los gobiernos deberían convocar siempre a sus ciudadanos cuando haya que tomar decisiones importantes para sus vidas.	4.11	1.1	0.509	0.557	0.063	0.124	0.611
*Governments should always convene their citizens when important decisions for their lives have to be made.*							
18. Es necesario que todas las personas participen de los problemas de su comunidad.	4.21	0.92	0.391	0.605	0.097	0.018	0.544
*It is necessary that all people participate in the problems of their community.*							

English items in italic. The factor loadings corresponding to the grouping factor have been highlighted in bold.

#### Belief in a just world

To assess this construct we used the Global Belief in a Just World Scale (Lipkus, 1991; Barreiro et al., 2014). The scale consists of seven items (e.g., “I believe that people get what they deserve,” “I believe that rewards and punishments are administered fairly”). The response format was Likert-type with five anchors (from 1 = “Strongly disagree” to 5 = “Strongly agree”). The scale showed adequate reliability (α = 0.83) and validity indicators (*S-B X2/_(df)_* = 2.98; *CFI* = 0.96; Δ^2^ = 0.96; *RMSEA* = 0.08). Higher scores indicate higher levels of belief in a just world.

#### Social dominance orientation

To assess this construct we used the reduced version of the SDO scale adapted and validated in Spain (Pratto et al., 2013), based on the of the original one (Pratto et al., 1994; Sidanius and Pratto, 1999). The scale comprises by four items (e.g., *“*We should not push for group equality,” “Group equality should be our ideal,” “Superior groups should dominate inferior groups”). The internal consistency of the scale (α = 0.82) and construct validity (CFI = 0.97; RMSEA = 0.042) was adequate. The response format follows a scale from: *1 = “Strongly disagree” to 5 = “Strongly agree*.” Higher levels suggest a greater social dominance orientation.

#### Socioeconomic level

The SES was measured by asking the family monthly income and the cohabitants partners’ educational level (Tabachnick and Fidell, 2007). The family monthly income was reported by participants ranging from 1 (“<500 euros per month”) to 8 (“>5,000 euros per month”) and the cohabitants partners’ educational level was categorized into six levels ranging from 1 (illiteracy) to 6 (postgraduate education). Based on the report of the Statistical National Institute -INE- (INE, 2019), 4,25% of participants belonged to the lower class, 23,15% to lower middle class, 66,01% to middle and 6.59% to upper middle.

#### Socio-demographic information questionnaire

We included questions to collect this type of information, including sex and age.

### Procedure and data analysis

We invited the subjects to participate in the online research voluntarily and requested their informed consent. The study was a first approach in order to preliminarily test the psychometric properties of the Social Justice scale. We informed them the research data would be used exclusively for academic-scientific purposes, preserving their anonymity. A geolocated survey service was used through social media (Facebook, Instagram and Twitter) and the survey took place between May and July of 2021. An IP registration control was used to ensure that only one questionnaire could be answered per person. Also, an email address was shared to resolve any research-related questions. No cases were dismissed from the total sample due to missing values, according to Tabachnick and Fidell (2007) criteria (>5%). We used IBM Statistical Package for the Social Sciences, Version 22 software (Lizasoain and Joaristi, 2003) and EQS Structural Equation Modeling Software, Version 6.4 (Bentler, 2007) for the statistical analyses. First, we examined the descriptive analyses for each item of the Social Justice scale, in addition to the corresponding reliability and validity assessments. Secondly, we conducted a path analysis to test a theoretical model incorporating the effects of social dominance and the belief in a just world.

## Results

### Constructing the scale of social justice representations

First, we analyzed the distribution of the 30 items to evaluate the three dimensions of Social Justice. We discarded 12 items for kurtosis excess according to the parameters proposed by Botella et al. (Botella et al., 1993), where is stated that values between −1,4 and 1,4 are adequate. Although all items respond directly to one of the dimensions of the construct, some of them obtained biased responses. For instance, when asked indirectly about the dimension Redistribution with items such as “The State should not give financial aid to anyone,” the vast majority of participants showed a broad disagreement. For Recognition dimension, items such as “Immigrants should adapt to the country they arrive in if they want to be accepted” were dismissed. Also, for Representation dimension “The citizens’ vote is often overestimated” or “Sometimes it is better for a few people with extensive political knowledge to have the right to vote, rather than for anyone to be able to do so.” The Table 1 shows the final 18 item’s, the internal consistency of each factor evaluated through Cronbach’s alpha coefficient, and the mean, standard deviation, item-total correlation, and alpha if the item is removed from the scale. In addition, the factor loadings for each item (KMO = 0.877; Bartletts test: *p* < 0.001) were informed.

All items present adequate psychometric indicators. The Representation dimension can improve its reliability by removing item 9, however it refers to an important aspect of the construct: interest to take part in political decisions, so the item has been kept. Subsequently, we performed a structural equation analysis to compare a unidimensional theoretical model with a model of three correlated dimensions. All indicators show a better data adjustment to the three correlated dimensions model, therefore, the unidimensional model should be discarded to analyze the representations of social justice with the tested items (Table 2).

**Table 2:** Confirmatory factor analysis of the social justice scale (SJS).

	*S-B X^2^_(gl)_*	*ΔS-B X^2^_(gl)_*	*CFI*	*IFI*	*RMSEA*
SJS (one dimension)	357.26 *_(135)_*	2.64	0.81	0.82	0.063 (0.055–0.070)
SJS (three dimension)	238.69 *_(132)_*	1.80	0.92	0.93	0.044 (0.035–0.053)

Adequate values: ΔS-B χ^2^_(gl)_ ≤ 3; NNFI, CFI, IFI ≥ 0.90; RMSEA ≤ 0.05.

Regarding the differences according to gender, we observed statistically significant differences in two of the three dimensions of social justice. In Recognition, women (*M* = 23.29; *SD* = 4.10) obtained higher scores (*t* = −2.91; *p* < 0.001; *Cohen’s d* = 0.26) with respect to men (*M* = 22.13; *SD* = 4.78), as did Representation, where women (*M* = 25.06; *SD* = 3.35) had higher levels (*t* = −3.82; *p* < 0.001; *Cohen’s d* = 0.34) than men (*M* = 23.79; *SD* = 4.12). There were no statistically significant differences in Redistribution. Regarding the factorial invariance of the questionnaire according to gender (Men: χ^2^ = 442.88, df = 135; CFI = 0.93; RMSEA = 0.04 [0.038–0.051]; Women: χ^2^ = 293.44, df = 135; CFI = 0.94; RMSEA = 0.05 [0.041–0.059]), the results on S-B Scaled Difference χ^2^ = 14.36, df = 15; (*p* = 0.50) allow us to assume both form invariance and invariance of equal factor loadings considering gender.

### Social dominance, belief in a just world and the social justice dimensions

Having arrived at a preliminary valid and reliable scale for assessing social justice, we proceeded to analyze the relationships between the three dimensions of the construct with two psychosocial variables: Social Dominance Orientation (SDO) and the Belief in a Just World (BJW) (Table 3).

**Table 3:** Relations between the social justice scale dimensions and psychosocial variables.

	1	2	3	4	5
1. Redistribution	0.73	0.429^**^	0.420^**^	−0.319^**^	−0.330^**^
2. Recognition		0.73	0.478^**^	−0.426^**^	−0.272^**^
3. Representation			0.65	−0.322^**^	−0.294^**^
4. Social dominance				0.82	0.401^**^
5. Belief in a just world					0.83

** *p <* 0.001.

Cronbach’s Alpha in the diagonal.

The psychosocial variables that more strongly correlates with social justice dimensions are Social Dominance with Recognition (*r* = −0.426; *p* < 0.001), and Participation (*r* = −0.322; *p* < 0.001), and the Belief in a Just World with Redistribution (*r* = −0.330; *p* < 0.001).

Finally, observing the significant and highly correlated relationships between Social Dominance and Belief in a Just World with the three dimensions of Social Justice, we tested a path analysis to account for influential relationships between the proposed theoretical variables (Supplementary Figure S1).

The metric indicators show an adequate data adjustment to the proposed theoretical model (S-B *X*^2^ = 24,47; *df* = 5; *ΔS-B X^2^(df)* = 4.89; *CFI* = 0.96; *RMSEA* = 0.037). Thirty-one percent of the Social Justice variance is explained by the influence of Group Dominance and Belief in a Just World (*R*^2^ = 0.37).

## Discussion

The main purpose of this work was to analyze preliminary the psychometric properties of the Social Justice Scale, whose dimensions represent the three dimensions of the construct proposed by (Fraser, 2008). We found reliability and validity indicators suitable for its use across our online sample, providing a first approach to the subject, making further exploration of the phenomenon possible.

One of the main discussions regarding the evaluation of the construct was whether Social Justice dimensions could be considered independently or as a single factor consisting of different dimensions. In this sense, our findings indicated that, while significantly correlated, they are independent and the items cannot be analyzed in a single dimension of the construct. The factorial invariance of the questionnaire according to gender has shown results which allow us to assume both an invariance of form and an invariance of equal factor loadings for men and women. However, it is suggested to continue studying the structure of the construct in other larger studies, based on this validated version of the scale. The use of a three-dimensional model of social justice (Fraser, 2008) in social contexts may be a key instrument to understand more deeply the origin of certain existing injustices with diverse causes. This can help different socio-educational professionals to find specific ways to reduce the levels of injustice related to each dimension with a higher prevalence.

We also inquired about the differences in each dimension of social justice by gender of the participants. Women in this study obtained higher scores in the Recognition and Representation dimensions, while Redistribution remained constant. These results are consistent with previous studies (Murillo et al., 2014), showing the greater sensitivity or critical awareness of women related to attitudes towards global social justice. However, when analyzing social justice through its three dimensions, the differences have not always been significant for redistribution and representation. In this sense, recognition affects aspects that are directly related to respect, tolerance and empathy towards diversity, compared to redistribution and participation (economic and political justice) which are dimensions in which leadership has traditionally been exercised by men. This aspect may be due to the fact that, although in recent decades the recognition and participation of women in the political sphere have increased, there are still significant levels of inequality with respect to men. In addition, recent studies indicate women have a greater tendency to be prosocial than men in their social justice representation (Jacott et al., 2014; Albalá Genol and Guerra, 2020). In any case, it is recommended to replicate this preliminary study in samples with a greater gender diversity.

The relationships between the three dimensions of social justice with psychosocial variables traditionally operating as the ideological background that contributes to the perpetuation of social inequalities, such as social dominance orientation and belief in a just world, were also examined. The correlational results have shown that, in coherence with previous research (Sutton and Douglas, 2005), BJW and SDO might be operating as psychosocial variables with a certain predictability towards the justification of social inequality, and therefore showing negative relationships with the three dimensions of social justice. In particular, Redistribution is the one that has the most negative link with BJW, which shows that this variable has a clear link with the hypothesis of a just world (Lerner, 1980): each person always gets what they deserve and therefore the distribution of resources is not necessary. Regarding to Social Dominance, Recognition and Representation are the dimensions that show the most predominant negative relationships, which might be related to a more hierarchical orientation on who should participate socially, excluding minority groups. We found statistically significant relationships between the variables in each case and went a step further by proposing a theoretical model where both belief in a just world and social dominance act as predictor variables of the three dimensions of social justice. We consider that belief in a just world and social dominance provide a strong ideological background for the three dimensions of social justice.

While meeting the aims proposed in this study, the work has several limitations that should be addressed in future research. First, although the study had acceptable results, it is a preliminary study for validation of the Social Justice Scale; therefore, it is necessary to continue replicating its assessment in larger and more heterogeneous samples, and to control the balance in key variables such as SES, gender and age. For example, it was not possible to perform an invariance test according to SES because only one of the four categories (middle class) reached the adequate number of cases to perform this type of analysis (Tabachnick and Fidell, 2007) (*n* > 200). Future studies should analyze different evaluations of SES. Secondly, we developed an assessment of the three dimensions of social justice based on Fraser’s (Fraser, 2008) ideas; however, the author’s theoretical proposal is much broader than the scale items developed in this study can address. The predictor variables of social justice should also be considered, given we could observe that belief in a just world and orientation to social dominance are directly involved in the assessment of each dimension of the social justice construct. Other variables such as openness to experience, need for cognitive closure, authoritarianism, ideological positioning, among others, may also contribute to the values of social justice. Besides, for future studies of the Social Justice Scale, it is suggested include the cultural factor considering the possibility of including native speakers and second language speakers, because no case has been considered so far. Also, we need to continue evaluating psychological variables that may be directly influenced by the construct, such as different expressions of prejudice, democratic regime support, local economic perception, as well as different citizen action forms. Also, future investigations should delve in the study of the concurrent and discriminant validity of the scale. A challenge for future researches, is to determine the origin of the correlation between low social justice representations (redistribution, recognition and representation) and other social attitudes, prejudice and behaviors that may result. The evidenced model is based on a dynamic perspective of social justice concept, never definitive or complete and is always open to reflection and improvement (Miller, 1999). In addition, the findings should be implications for future studies related to social justice, since a unidimensional analysis may lead to errors in its evaluation and in its possible relationship with other psychosocial variables.

As proposed by (Westheimer, 2015), it is essential to pursue the discussion on the importance of developing a citizenship-oriented social justice that considers citizens to be fundamental active change agents in reproducing the inequities and injustices of the world we live in. A possible perspective for future studies could be to consider the incorporation of relevant dimensions for younger citizenship and not directly addressed by redistribution, recognition and representation, for example: environmentalism, feminism and digital citizenship.

## Data availability statement

The raw data supporting the conclusions of this article will be made available by the authors, without undue reservation.

## Author contributions

EE and AR: substantial contributions to the conception or design of the work and the acquisition, analysis or interpretation of data for the work. EE, AB, and MG: acquisition, analysis, or interpretation of data for the work and drafting the work or revising it critically for important intellectual content. All authors agree to be accountable for all aspects of the work in ensuring that questions related to the accuracy or integrity of any part of the work are appropriately investigated and resolved. All authors contributed to the article and approved the submitted version.

## Conflict of interest

The authors declare that the research was conducted in the absence of any commercial or financial relationships that could be construed as a potential conflict of interest.

## Publisher’s note

All claims expressed in this article are solely those of the authors and do not necessarily represent those of their affiliated organizations, or those of the publisher, the editors and the reviewers. Any product that may be evaluated in this article, or claim that may be made by its manufacturer, is not guaranteed or endorsed by the publisher.
