# Supplementary material for: Assessment of social justice dimensions in young adults: The contribution of the belief in a just world and social dominance orientation upon its rising
Source: Front Psychol. 2022 Nov 3;13:997423. doi: 10.3389/fpsyg.2022.997423 (PMC9670803; doi:10.3389/fpsyg.2022.997423)
Supplement: Supplementary file 1 [file Image_1.pdf]

Supplementary Material

Supplementary Figures

Figure 1.

Path analysis between Group Dominance, Belief in a Just World, Dimensions of Social Justice, and Support for Intergroup Equality.

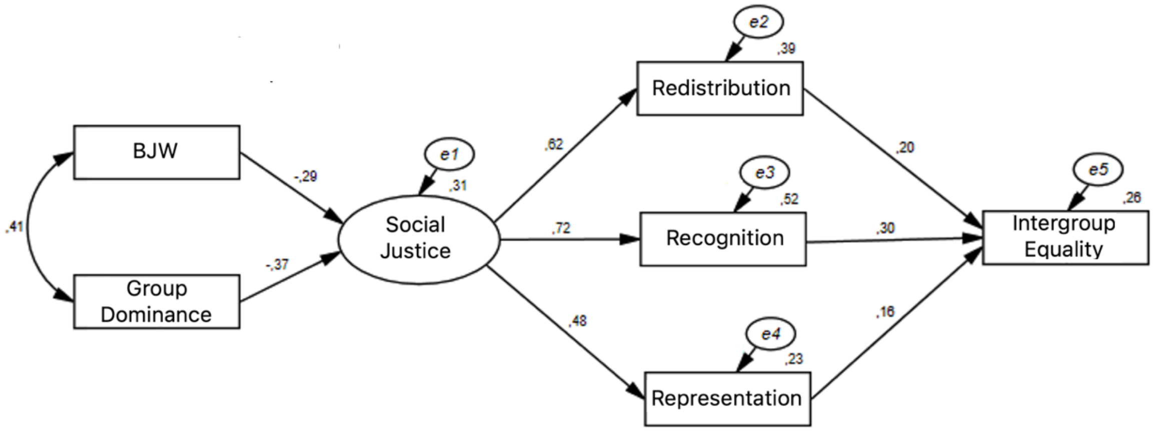

Note. All parameters has statistical significance at p < .001. Model fit: S-B X^2 = 28,61; df = 6; ΔS-B $X^2(df)$ = 4.76; CFI = .94; RMSEA = .04.
